# Chemical Composition of Clove and Fennel Seed Essential Oils and a Comparison of Their *In Silico* and *In Vitro* Antibacterial Activity with That of Their Main Compounds

**DOI:** 10.3390/cimb47090694

**Published:** 2025-08-27

**Authors:** Achraf Abdou, Fatima Ezzahra Maaghloud, Fatima Zahra Kamal, Said Rammali, Alin Ciobica, Vasile Burlui, Cristina Albert, Abdelhakim Elmakssoudi, Bogdan Novac, Mohamed Dakir

**Affiliations:** 1 Laboratory of Organic Chemistry, Materials, Electrochemistry, and Environment, Faculty of Sciences Ain Chock, Hassan II University, Casablanca 20000, Morocco; fatimaghloud@gmail.com (F.E.M.); h.elmakssoudi@gmail.com (A.E.); dakir_m@yahoo.fr (M.D.); 2 Higher Institute of Nursing Professions and Health Technical (ISPITS), Casablanca 20100, Morocco; fatimzahra.kamal@gmail.com; 3 Laboratory of Agro-Alimentary and Health, Faculty of Sciences and Techniques, Hassan First University of Settat, B.P. 539, Settat 26000, Morocco; said.rammali90@gmail.com; 4 Human Nutrition, Bioacives and Oncogenetics Team, Faculty of Sciences, Moulay Ismail University, Meknes 11201, Morocco; 5 Department of Biology, Faculty of Biology, “Alexandru Ioan Cuza” University of Iași, Carol I Avenue, No. 20A, 700505 Iași, Romania; alin.ciobica@uaic.ro; 6 “Olga Necrasov” Center, Department of Biomedical Research, Romanian Academy, 010071 Iasi, Romania; 7 “Ioan Haulica” Institute, Apollonia University, Pacurari Street 11, 700511 Iași, Romania; 8 CENEMED Platform for Interdisciplinary Research, “Grigore T. Popa” University of Medicine and Pharmacy of Iasi, 16th Universitatii Street, 700115 Iasi, Romania; 9 Clinical Department, Apollonia University, Păcurari Street 11, 700511 Iasi, Romania; secretariat@univapollonia.ro; 10 Faculty of Medicine, University of Medicine and Pharmacy “Grigore T. Popa”, 700115 Iasi, Romania; bogdannvc@gmail.com

**Keywords:** clove bud, seed of fennel, eugenol, estragole, antibacterial activity

## Abstract

This study aimed to assess the chemical composition and antibacterial potential of essential oils (EOs) from two plants: clove buds (*Syzygium aromaticum*) and fennel seeds (*Foeniculum vulgare*) EOs. The major compounds, eugenol and estragole, were isolated from these oils and tested against *Escherichia coli*, *Staphylococcus aureus*, and *Pseudomonas aeruginosa*. The EOs were obtained via hydrodistillation and analyzed using Gas Chromatography–Mass Spectrometry (GC-MS). Clove oil was found to be rich in eugenol (68.51%), while fennel seed oil was dominated by estragole (93.30%). Antibacterial activity, assessed by the agar disc diffusion method and supported by MIC/MBC testing, revealed that eugenol exhibited the highest efficacy, with MIC values ranging from 0.58 to 1.15 mg/mL and MBC values from 1.15 to 2.30 mg/mL, particularly against *S. aureus* and *P. aeruginosa*. *In silico* analysis was conducted to evaluate pharmacokinetics, toxicity, and molecular docking interactions. ADME predictions indicated good oral bioavailability and high membrane permeability for both compounds, with eugenol displaying superior solubility and better compliance with Lipinski’s Rule of Five. Molecular docking simulations confirmed the antibacterial potential, with eugenol showing stronger binding affinities to bacterial targets (−7.8 kcal/mol), forming more stable and diverse interactions compared to estragole. However, toxicity predictions indicated potential mutagenic, carcinogenic, and cardiotoxic (hERG inhibition) risks for both compounds.

## 1. Introduction

Aromatic and medicinal plants have long been recognized for their ability to produce a wide variety of bioactive secondary metabolites, among which essential oils (EOs) are particularly valued. These oils are volatile at room temperature, hydrophobic, and can be extracted from various plant parts such as flowers, leaves, roots, bark, fruits, and seeds [1,2]. Chemically, EOs are complex mixtures rich in volatile compounds like terpenes, terpenoids, and phenylpropenes [3,4]. Owing to their distinctive flavors and fragrances, EOs are widely utilized in the food, cosmetic, pharmaceutical, and aromatherapy industries [5,6,7].

Beyond their sensory properties, essential oils have attracted scientific interest for their wide range of biological activities, notably their antimicrobial potential. Their natural origin, low toxicity, biodegradability, and cost-effectiveness make them appealing alternatives to synthetic antimicrobials, particularly in the context of increasing antibiotic resistance [8,9]. The antibacterial effects of EOs are often attributed to their major constituents, which can disrupt microbial membranes, inhibit metabolic enzymes, and interfere with cell signaling mechanisms [10,11].

Among the most studied EO components are phenylpropenes, especially eugenol and estragole, which occur in various medicinal and aromatic plants (MAPs). Eugenol (C_10_H_12_O_2_) is a well-known phenolic compound widely present in essential oils, with *Syzygium aromaticum* (clove) being its principal natural source [12,13]. It exhibits a range of biological properties, including strong antimicrobial, antioxidant, and anti-inflammatory activities. On the other hand, estragole (C_10_H_12_O), also known as methyl chavicol or p-allylanisole, is the predominant compound in the essential oil of *Foeniculum vulgare* (fennel), a plant widely used in traditional medicine and culinary applications [14,15].

**Figure 1:**
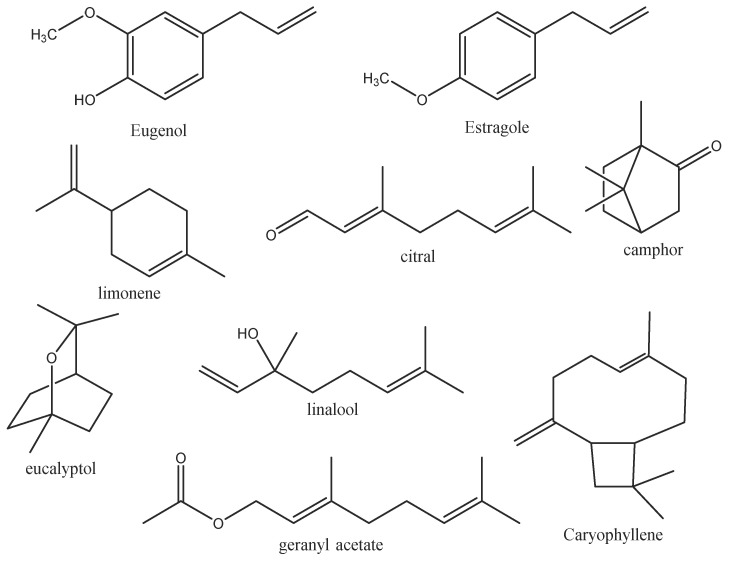
Chemical structure of some bioactive compounds in essential oils.

Essential oils (HEs) are complex mixtures of bioactive compounds, including monoterpenes, sesquiterpenes, phenylpropanoids, aldehydes, alcohols, esters, ketones, and terpenoids. Key compounds like limonene, caryophyllene, eugenol, estragole, citral, linalool, geranyl acetate, camphor, and eucalyptol (Figure 1) contribute to the oils’ antimicrobial, anti-inflammatory, and antioxidant properties, making them valuable in both traditional and modern medicine for a wide range of therapeutic effects. While whole essential oils are often tested for their antibacterial properties, the specific contributions of their individual constituents remain unclear. Isolating and studying major compounds like eugenol and estragole allows for a more precise understanding of their roles and mechanisms in antimicrobial action, helping to determine whether these compounds act synergistically or independently of the whole oil matrix [16].

This study focuses on the extraction of essential oils from two plants: clove buds and fennel seeds, and the analysis of their chemical compositions. Additionally, the major compounds of these essential oils were isolated. The antibacterial activity of the extracted essential oils and their main compounds was evaluated against three bacterial strains.

The objective of this study was to compare the antibacterial activity of essential oils (HE) and their main compounds to determine if the isolated compounds exhibit higher efficacy than the crude oils themselves. Additionally, the activity of isolated compounds was compared, and the findings were validated through *in silico* studies, with the goal of exploring their potential as natural antibacterial agents.

## 2. Materials and Methods

### 2.1. Sourcing and Preparation of Material

Clove buds and fennel seeds were sourced from a spice market in Casablanca, Morocco, for essential oil extraction. The 100 g of plant materials were dried, ground into a fine powder, and extracted via hydrodistillation over a period of 4 h. All organic reagents and solvents used in this study were of analytical grade and were sourced from Sigma-Aldrich (St. Louis, MO, USA) and Merck (Darmstadt, Germany), ensuring high purity and reliability for the experimental procedures. The extracted products were purified using column chromatography on silica gel, with hexane-ethyl acetate as the eluent. The gas chromatography was conducted with a Shimadzu 2010 GC coupled to a Shimadzu QP-2010 mass spectrometer (Shimadzu Corporation, Kyoto, Japan) operating at an ionization voltage of 70 eV. The oven temperature was programmed to increase from 80 °C to 200 °C at a rate of 3 °C/min, followed by a ramp to 280 °C at 15 °C/min, held for 5 min. Helium was used as the carrier gas at a flow rate of 1.27 mL/min and a linear velocity of 31.7 cm/s [17].

### 2.2. Antibacterial Assays

The antibacterial activity of three compounds against *Escherichia coli* (ATCC 25922), *Staphylococcus aureus* (ATCC 43300), and *Pseudomonas aeruginosa* (ATCC 27853) was determined using the agar disc diffusion technique on Mueller Hinton agar. A 100 μL aliquot of newly generated inoculum (10^8^ bacterial cells/mL, according to the McFarland standard) was equally distributed over sterile Mueller Hinton agar plates. Filter paper discs (approximately 6 mm in diameter) were wet with 15 µL of each plant EO, and major compounds were dissolved in DMSO and dropped onto the agar plates, pushing them down to achieve full contact. The plates were then incubated at 37 °C for 24–72 h. Following incubation, the inhibition zones were measured in millimeters. DMSO and amoxicillin (30 µL) were used as negative and positive controls, respectively. All the tests were performed in triplicate to ensure reproducibility [18].

### 2.3. Microdilution Test

The minimum inhibitory concentration (MIC) and minimum bactericidal concentration (MBC) of the oils and their main compounds were determined using the microdilution method on 96-well plates, following standard protocols. Each well was filled with 50 µL of Mueller Hinton broth, and varying concentrations of the product dissolved in 10% DMSO (dimethyl sulfoxide) were added. Then, 50 µL of a bacterial suspension, adjusted to 10^6^ CFU/mL, was introduced into all wells. The microplates were incubated at 37 °C for 24 h. Microbial growth in each well was assessed using the Resazurin assay. To each well, 30 μL of a 0.01% (*w*/*v*) Resazurin solution was added, and the plates were incubated for an additional 2 h at 37 °C. Growth was indicated by a color change from purple to pink [19].

For each microplate, the following control columns were included:

A column containing Mueller Hinton medium and bacterial solution.

A column containing Mueller Hinton medium, bacterial solution, and DMSO.

A column containing only Mueller Hinton medium.

### 2.4. Statistical Analysis

The student’s *t*-test was used to establish statistical significance between means. One-way ANOVA with SPSS software version 20. [* *p* < 0.05; ** *p* < 0.01; *** *p* < 0.001] was used to examine differences between groups [20]. 

### 2.5. Isolation of Eugenol and Estragole

Clove bud essential oil underwent a chemical treatment procedure to extract eugenol. Using hexane-ethyl acetate (80/20) as the eluent, column chromatography on silica gel was used to purify estragole.

### 2.6. Product Characterization

Eugenol

^1^H NMR (300 MHz, CDCl_3_) δ (ppm): 6.89 (d, *J* = 8.5 Hz, 1H), 6.72 (dd, *J* = 8.5, 2.4 Hz, 1H), 6.71 (d, *J* = 2.5 Hz, 1H), 5.99 (ddt, *J* = 16.8, 10.0, 6.7 Hz, 1H), 5.74 (s, 1H), 5.16–4.98 (m, 2H), 3.87 (s, 3H), 3.35 (dt, *J* = 6.6, 1,4 Hz, 2H). ^13^C NMR (75 MHz, CDCl_3_) δ 146.64, 144.04, 138.02, 132.06, 121.32, 115.65, 114.50, 111.82, 55.97, 40.02. IR υ (cm^−1^): 3520 (O-H) 2976 (C=CH-Ar), 2841 (CH=CH_2_), 1612 (C=C-Ar), 1260 (Ar-C-O). MS: *m*/*z* = 164 [12].

Estragole

^1^H NMR (300 MHz, CDCl_3_) δ (ppm): 7.18 (d, *J* = 8.6 Hz, 2H), 6.92 (d, *J* = 8.7 Hz, 2H), 6.03 (ddd, *J* = 16.9, 10.1, 6.7 Hz, 1H), 5.18–5.09 (m, 2H), 3.84 (s, 3H), 3.41 (dt, *J* = 6.6, 1.5 Hz, 2H). ^13^C NMR (75 MHz, CDCl_3_) δ (ppm): 158.19, 138.08, 132.24, 129.68, 115.58, 114.01, 55.35, 39.52. IR υ (cm^−1^): 2995 (C=CH-Ar), 2831 (CH=CH_2_), 1612 (C=C-Ar), 1257 (Ar-O-C). MS: *m*/*z* = 148.

### 2.7. ADMETox

ADME (Absorption, Distribution, Metabolism, and Excretion), and drug-likeness of two ligands, eugenol and estragole, was performed using the PreADMET online platform (https://preadmet.webservice.bmdrc.org accessed on 10 March 2025). 

Drug-likeness prediction was performed using several established *in silico* filters, notably Lipinski’s Rule of Five, which correlates molecular properties such as molecular weight, hydrogen bond donors and acceptors, and lipophilicity with oral bioavailability [21]. Additionally, we applied the Lead-like rule, derived from structural comparisons between lead compounds and marketed drugs, which is useful for identifying chemically tractable starting points in drug development [22]. The PreADMET platform incorporates these rules into a comprehensive drug-likeness module to help assess the compounds’ potential as oral drug candidates.

For ADME prediction, we used *in silico* simulations to estimate intestinal absorption and permeability. Caco-2 and MDCK cell models, which simulate the human intestinal barrier and epithelial transport, respectively, were employed to predict permeability and uptake. We also used Human Intestinal Absorption (HIA) models to estimate oral bioavailability and skin permeability models to evaluate potential for transdermal delivery [23].

Toxicity prediction was carried out using PreADMET’s toxicity module. *In silico* toxicity models are increasingly important in early drug discovery, as a significant proportion (~30%) of candidate compounds fail due to safety concerns [24]. The tool provides reliable predictions of mutagenicity based on the Ames test (including bacterial strains TA100, TA98, and TA1535) and long-term rodent carcinogenicity models in rats and mice, enabling early identification of genotoxic or carcinogenic risks [25].

### 2.8. Molecular Docking

The chemical structures of the ligands, eugenol and estragole, were obtained from the PubChem database (https://pubchem.ncbi.nlm.nih.gov accessed on 2 March 2025). The three-dimensional (3D) structures of the target proteins were sourced from the Protein Data Bank (PDB) (https://www.rcsb.org accessed on 2 March 2025), with the selected strains and associated PDB IDs listed below (Table 1).

Initial preparation of the protein and ligand structures was conducted using Discovery Studio software. This involved the removal of water molecules, the addition of polar hydrogen atoms, and the assignment of Kuhlmann charges. The processed structures were then saved in PDBQT format for docking analysis. Docking simulations were carried out using AutoDockTools 1.5.6, which allowed for multiple docking runs to be performed efficiently. To ensure all potential ligand binding sites were thoroughly examined, the docking grid was set to cover the entire protein interaction region [26].

## 3. Results

### 3.1. Chemical Part

We carried out the extraction of clove bud essential oil using two hydrodistillation methods: simple hydrodistillation and Clevenger hydrodistillation, while maintaining the same quantity of cloves and extraction duration. All experiments were performed in triplicate. Table 2 below presents the obtained results.

**Figure 2:**
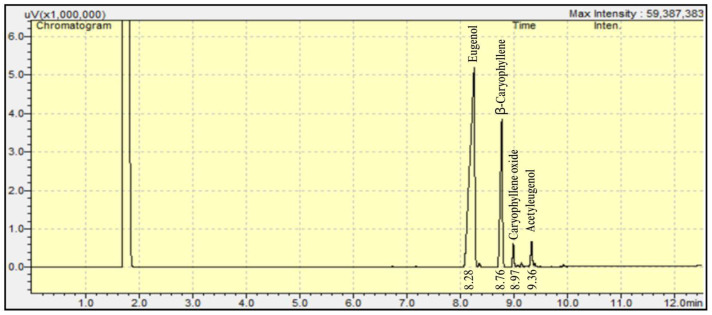
Chromatogram of clove essential oil (t = 13 min).

Clove essential oil was examined by GC-MS. Four constituents accounting for 97.88% of the total oil were identified (Figure 2). It is a chemotype essential oil, with eugenol as the predominant compound at 68.51%, followed by β-caryophyllene at 16.93%, eugenol acetate at 10.35%, and caryophyllene oxide at 2.09% (Table 3).

The purification of the primary compound in clove essential oil was achieved via chemical treatment, resulting in a 96% purity yield as determined by gas chromatography. Eugenol was separated from the oil by converting it into eugenolate salt with the addition of sodium hydroxide (NaOH), which made it soluble in the aqueous layer. The eugenolate was then hydrolyzed back to eugenol by adding sulfuric acid (H_2_SO_4_) to create an acidic environment (pH = 1). Eugenol was subsequently extracted from the aqueous layer using dichloromethane with a yield of 98%. The purified eugenol, a yellowish liquid, was verified through FT-IR spectroscopy, GC/MS, and ^1^H and ^13^C-NMR analyses (as shown in supplementary Figure S1–S4) [28].

**Figure 3:**
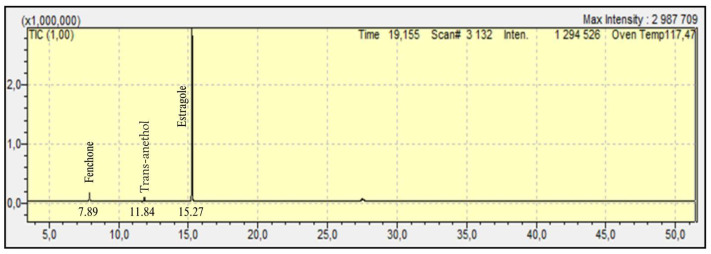
Chromatogram of fennel seeds essential oil (t = 53 min).

A Clevenger apparatus was utilized to prepare the fennel seeds for hydrodistillation, which is the method of essential oil extraction. The EO was extracted with a yield of 3%. The oil is a liquid that crystallizes around 4 °C, ranging from colorless to extremely light yellow, with a pronounced aniseed fragrance reminiscent of fennel. The EO was analyzed using GC-MS. The chromatogram showed three peaks for oxygenated monoterpenes: fenchone, anethole, and estragole. This investigation demonstrates that fennel seed essential oil is chemotyped, with estragole accounting for 93.30% (Figure 3 and Table 4).

Estragole was extracted from fennel seed essential oil using column chromatography over silica gel and n-hexane/ethyl acetate combinations with a yield of 90%. Pure estragole was a colorless to yellowish liquid. It was thoroughly characterized using multiple spectroscopic techniques, including GC-MS, IR spectroscopy, ^1^H NMR, and ^13^C NMR, as shown in Figures S5–S8.

### 3.2. Biological Part

The results showed that each substance tested inhibited bacterial growth to varying degrees. Table 5 and Table 6 show the three strains; *S. aureus*, *E. coli*, and *P. aeruginosa*, together with their inhibition diameters, minimum bactericidal concentrations (MBCs), and minimum inhibitory concentrations (MICs). The three tested strains were sensitive to the EOs and their main compounds, with diameters ranging from 7.00 to 18.65 mm. Fennel seed essential oil had the shortest diameters of the samples, measuring 9.50 mm for *S. aureus*, 9.67 mm for *E. coli*, and 7.00 mm for *P. aeruginosa*. Clove bud essential oil exhibited a high activity against *S. aureus* and *E. coli*, with diameters of 15 mm for both, but was less efficient against *P. aeruginosa*, with a diameter of 11.33 mm. Eugenol and estragole, as purified molecules, have stronger activity than their respective essential oils (Table 5). The MIC values corroborated with the aromatogram test. Eugenol had the lowest MIC values for *S. aureus* and *P. aeruginosa*, at 0.58 mg/mL and 2.32 mg/mL, respectively. The primary ingredient in the essential oil has an MBC value of 9.2 mg/mL against *S. aureus* and *P. aeruginosa*. In comparison to the other samples, estragole had the most effective MIC (2.30 mg/mL) and MBC (14.50 mg/mL) values against *E. coli* (Table 6). Eugenol displayed the broadest zone of sensitivity against the strains, owing to its high lipophilicity, which allows it to penetrate bacterial cell walls. This penetration alters the fluidity and permeability of the cell membrane, eventually resulting in bacterial malfunction and lysis.

To determine whether the antibacterial effect is bactericidal (killing bacteria) or bacteriostatic (inhibiting bacterial growth), the MBC/MIC ratio is calculated. A ratio of ≤4 indicates a bactericidal effect, meaning the substance kills bacteria at a concentration close to that which inhibits growth, while a ratio > 4 suggests a bacteriostatic effect, where the substance inhibits bacterial growth at much lower concentrations than required to kill the bacteria. For example, eugenol exhibited an MBC/MIC ratio of 15.86 against *S. aureus*, indicating a bacteriostatic effect, and a ratio of 3.97 against *P. aeruginosa*, indicating a bactericidal effect. Estragole displayed ratios greater than 4, suggesting it has a bacteriostatic effect against all tested strains. These ratios provide valuable insights into the mechanisms of action of the tested substances, enhancing the reliability and interpretability of the antimicrobial findings by distinguishing between substances that merely inhibit bacterial growth and those that actively kill bacteria.

### 3.3. In Silico Study

#### 3.3.1. Drug-likeness Profiling

Eugenol and estragole were evaluated for their drug-likeness using various *in silico* filters. According to the CMC-like rule, eugenol qualified, whereas estragole did not due to its lower molecular weight. Both compounds complied with the Lead-like rule, which considers properties such as molecular weight ≤ 350 Da and logP ≤ 3. They also satisfied Lipinski’s Rule of Five, indicating favorable oral bioavailability. However, both violated two parameters under the MDDR-like rule, specifically the number of rings and rotatable bonds, which is why they were classified as “mid-structure.” Finally, only estragole fell within the 90% cutoff for the WDI-like rule, while eugenol did not, due to its high Balaban index (Table 7).

#### 3.3.2. ADME and Physicochemical Properties

Both compounds exhibited high human intestinal absorption (HIA), with estragole reaching 100% and eugenol reaching 96.77%. Eugenol showed greater BBB permeability (2.26 vs. 1.51). MDCK permeability was significantly higher for eugenol, reflecting superior membrane permeability. Solubility was higher for eugenol in both buffer and water. Both compounds bind strongly to plasma proteins (100%) and are CYP2C9 and CYP2C19 inhibitors, but not CYP3A4 or CYP2D6 inhibitors. Neither compound was a P-gp substrate or inhibitor, and skin permeability was within an acceptable range (Table 8).

#### 3.3.3. Toxicity and Ecotoxicity Predictions

Both compounds tested mutagenic in the Ames test and showed positive results in TA100 and TA1535 assays, indicating genotoxic potential. Carcinogenicity was observed in mice for both; however, only eugenol was carcinogenic in rats. Both had medium risk for hERG inhibition, suggesting moderate cardiotoxicity. Ecotoxicity results (algae, daphnia, medaka, minnow) showed low acute toxicity, with LC50 values in a similar range (Table 9).

#### 3.3.4. Molecular Docking Scores

Eugenol consistently demonstrated the highest binding energies (stronger affinities) than estragole across all bacterial proteins, with top scores of −7.8 kcal/mol for *S. aureus* (1QME) and *P. aeruginosa* (8AID). Estragole also showed good binding, but weaker in comparison (Table 10).

## 4. Discussion

The aims of the present study are to evaluate the essential oils (EOs) chemical composition and antibacterial efficacy, extracted from clove buds (*Syzygium aromaticum*) and fennel seeds (*Foeniculum vulgare*), along with their respective primary constituents, eugenol and estragole. The results provide compelling evidence of the bioactive potential of these natural compounds, reinforcing the essential oils’ roles and their isolated constituents as promising antibacterial agents.

We obtained clove EO via Clevenger-type hydrodistillation with a yield of 12%, and the major constituent was eugenol (68.51%), followed by β-caryophyllene, acetyleugenol, and caryophyllene oxide. The fennel seed EO had a low yield of 3% and was a chemotype oil dominated by estragole (93.30%). These results are in agreement with previous literature that reports high levels of eugenol in *S. aromaticum* and high levels of estragole in *F. vulgare*, indicating both species have geographic chemotypic characteristics [29,30].

The antibacterial test revealed that both EOs and their isolated components were active against *Escherichia coli*, *Staphylococcus aureus*, and *Pseudomonas aeruginosa*, with varying degrees of efficacy. Eugenol had the highest inhibition zones and the lowest MIC/MBC values of the samples tested, particularly against *P. aeruginosa* and *S. aureus*. The higher activity can be associated with the well-established mechanism of eugenol action, including bacterial membrane disruption due to its phenolic structure and its high lipophilicity. Moreover, eugenol’s membrane-disruptive action is not limited to a single bacterial class; however, Gram-positive bacteria such as *S. aureus* generally exhibit higher susceptibility compared to Gram-negative bacteria like *E. coli* and *P. aeruginosa*. This differential susceptibility is largely attributed to the outer membrane of Gram-negative bacteria, which acts as an additional barrier to hydrophobic molecules like eugenol. Many studies have reported eugenol’s antimicrobial effect. Özel et al. [31] reported that eugenol, carvacrol, and citronellol have strong antimicrobial activity, with eugenol exhibiting superior efficacy against *Staphylococcus aureus* and *Pseudomonas aeruginosa*. The mode of action of these compounds involves disrupting bacterial membranes, resulting in changes to surface hydrophobicity, charge, and overall membrane integrity [32,33]. EO components have also been shown to display synergistic interactions with conventional antibiotics, thereby elevating the effect of the antibiotic while reducing the dose [31]. It has also been found that Gram-positive bacteria like *S. aureus* have higher susceptibility to EOs compared to Gram-negative bacteria such as *E. coli* and *P. aeruginosa* [34].

Estragole was also active, but it was weaker against bacteria, most notably *P. aeruginosa*, and thus suggested a limited antimicrobial spectrum, and likely an entirely different mechanism of interaction with the bacterial cells. Song et al. and Bezerra et al. [35,36] also reported that estragole shows direct antibacterial activity, but in limited forms against a range of bacteria, including *Pseudomonas syringae pv. actinidiae* and *Staphylococcus aureus*.

Our results differ slightly from the literature, which can be significantly influenced by factors such as climate, soil conditions, and geographic location, all of which affect the types and concentrations of bioactive compounds in the oils. For instance, clove EOs from different regions have been reported to contain varying levels of eugenol, a key compound responsible for its antibacterial activity, depending on the local growing conditions. Similarly, fennel essential oil’s content of estragole or anethole, which plays a major role in its antimicrobial activity, can vary based on geographical factors. Moreover, extraction methods, such as steam distillation versus cold pressing, can also affect the yield and quality of essential oils, further influencing their antibacterial efficacy. These variations highlight the importance of considering environmental and methodological factors when interpreting the antibacterial potential of essential oils.

An important observation is that the isolated compounds, eugenol and estragole, often show enhanced antibacterial activity relative to their crude essential oils. This phenomenon could be due to the presence of minor components within the oils that exert antagonistic or dilutive effects on the major active compounds, thereby reducing overall efficacy [37]. Essential oils are complex mixtures, and interactions among constituents, whether synergistic or antagonistic, significantly influence their bioactivity. Such interactions underscore the need for further detailed combinatorial and dose-dependent studies to optimize the therapeutic potential of essential oils and their constituents [31].

Eugenol showed the strongest antibacterial activity among the tested compounds, with the lowest MIC and MBC values, particularly against *S. aureus* and *P. aeruginosa*. Its efficacy is attributed to its phenolic structure and membrane-disrupting properties. Estragole exhibited moderate activity, indicating a narrower antimicrobial spectrum. Clove essential oil (EOC) performed better than expected, especially against *P. aeruginosa*, suggesting synergistic effects among its constituents [38,39]. In contrast, fennel essential oil (EOF) was the least effective across all strains. Gram-positive *S. aureus* was more susceptible than Gram-negative bacteria. Overall, pure compounds were more active than crude oils. These results support eugenol’s pharmaceutical potential as a natural antimicrobial agent [40,41]. Our MIC and MBC values are consistent with previous reports but also highlight some strain-specific differences. For instance, the MIC of eugenol against *S. aureus* (0.58 mg/mL) aligns with studies by Marchese et al. [42] and Ribeiro-Santos et al. [43], which reported MIC values ranging from 0.5 to 1 mg/mL for similar Gram-positive bacteria. In contrast, higher MIC values were observed for *E. coli* and *P. aeruginosa*, consistent with the inherent resistance of Gram-negative strains due to their protective outer membrane. The weaker antimicrobial performance of estragole (MIC: 2.30–4.60 mg/mL) supports previous findings that its bioactivity is limited compared to phenolic compounds like eugenol [44].

Furthermore, the data show that isolated compounds (eugenol and estragole) were generally more active than their respective essential oils. This suggests the presence of potential antagonistic interactions among EO constituents that may reduce the efficacy of the major components. Alternatively, some minor compounds may contribute to synergistic effects that enhance activity, as seen in the strong antibacterial effect of clove EO against *P. aeruginosa* (MIC: 0.50 mg/mL). Such interactions are well-documented in the literature, where essential oils often display increased antimicrobial activity when used in combination or when their components act on different microbial targets [45,46]. These findings underline the complexity of essential oil pharmacodynamics and the importance of studying both individual and combined effects for future therapeutic development.

The *in silico* drug-likeness profiles show that both eugenol and estragole hold promise as lead molecules, despite minor rule violations. Eugenol exhibits better compliance with CMC-like and WDI-like rules, while both compounds satisfy the Lipinski and Lead-like criteria. This indicates favorable bioavailability and drug development potential, especially for oral formulations.

The ADME profiles of the compounds support their pharmacological potential. Eugenol has superior BBB penetration, MDCK permeability, and aqueous solubility, which could translate into better CNS access and formulation properties. Both compounds show high intestinal absorption and enzyme selectivity, but the absence of interactions is especially favorable for avoiding multidrug resistance.

In terms of toxicity, the mutagenic and partially carcinogenic behavior of both molecules, particularly eugenol in rats, warrants caution. The moderate hERG inhibition risk must be addressed through chemical modification or dosage control to prevent cardiotoxic effects. However, their low aquatic toxicity highlights environmental safety at therapeutic levels.

The ligand–protein interaction diagrams for the bacterial species *E. coli* (PDB ID: 1F34, 4K3P, 5U10), *S. aureus* (PDB ID: 1QME, 3T05, 3WQT), and *P. aeruginosa* (PDB ID: 1IUV, 1RTT, 8AID) demonstrate distinct and species-specific binding patterns for the two ligands, estragole and eugenol. In *E. coli*, estragole forms hydrogen bonds with residues such as GLU C:21 and ASN C:212 in 1F34, interacts through polar contacts with ASN A:127 and ARG B:73 in 4K3P, and binds within a largely hydrophobic pocket in 5U10, involving HIS A:43 and LYS A:260. Eugenol exhibits similar but often more extensive interaction networks, especially in hydrogen bonding and van der Waals contacts. In *S. aureus*, estragole binds via hydrogen bonding and π–alkyl interactions in 1QME and forms π–sulfur and π–alkyl interactions with MET D:257 and LYS D:260 in 3T05. In 3WQT, estragole engages GLU A:299 and ASP A:239 through hydrogen bonds, while eugenol forms more diverse interactions, including π–cation, π–anion, and hydrogen bonds with ASP B:346, ASN B:267, and GLU C:35, indicating broader binding stabilization. Similarly, in *P. aeruginosa*, estragole predominantly binds through π–alkyl and π–sigma interactions (e.g., with HIS A:152 and PHE A:151 in 1IUV), while eugenol forms more stable complexes through additional hydrogen bonds with SER A:270 and ARG A:341. The 1RTT complex for both ligands shows strong contributions from ARG and GLU residues, with π–cation and hydrogen bonding playing central roles. In 8AID, estragole and eugenol share hydrogen bonding interactions with GLU B:18 and ARG A:9, but eugenol exhibits greater binding stabilization due to its additional polar contacts. Across all species, conserved residues such as ARG, GLU, ASP, and PHE are key contributors to ligand stabilization via hydrogen bonding and π-based interactions. These findings collectively highlight that both estragole and eugenol achieve stable binding through a combination of electrostatic and hydrophobic interactions, with eugenol generally displaying a slightly stronger and broader interaction profile. These results provide meaningful insights into ligand specificity and support their potential as structural templates for the development of new antibacterial agents (Table 11).

Several factors may account for the discrepancies between the *in silico* and *in vitro* findings. Firstly, the bioavailability and solubility of the compounds can significantly influence their antimicrobial efficacy. *In vitro*, the effectiveness of a compound depends not only on its binding affinity to bacterial targets but also on its ability to penetrate bacterial cell membranes and reach sufficient concentrations at the site of action. The presence of other compounds in the essential oils, which could exert synergistic effects, further complicates this relationship. For example, the combined action of multiple components in an essential oil may enhance or modulate the activity of the main antimicrobial compounds, which is not accounted for in molecular docking studies focused on individual compounds. These complex interactions within the EO matrix could explain why the *in vitro* results sometimes diverge from the *in silico* predictions. While molecular docking provides valuable insights into the potential interactions between antimicrobial compounds and bacterial proteins, the correlation with MIC/MBC values is not always straightforward. The bioavailability, solubility, and synergistic effects within the essential oils play crucial roles in determining the actual antimicrobial activity. Therefore, a holistic approach that combines both *in silico* and *in vitro* methods is essential for a comprehensive understanding of the antimicrobial mechanisms and the potential clinical application of these compounds.

Overall, both compounds show valuable drug-like characteristics, with eugenol offering a slight advantage due to its better permeability, solubility, and binding interactions. However, mutagenicity and carcinogenicity risks must be carefully managed in future development steps.

## 5. Conclusions

This study comprehensively investigated the antibacterial and pharmacokinetic potential of eugenol and estragole, the primary constituents of clove (68.51%) and fennel seed (93.30%) essential oils, respectively. Experimental analyses confirmed that both essential oils and their isolated compounds exhibited antibacterial activity against *Staphylococcus aureus*, *Escherichia coli*, and *Pseudomonas aeruginosa*, with eugenol showing superior efficacy, particularly against *S. aureus* and *P. aeruginosa*. Eugenol demonstrated the lowest MIC (0.58 mg/mL) and MBC (1.16 mg/mL) values among the tested samples, while estragole displayed higher MIC values (2.30–4.60 mg/mL), indicating a weaker antimicrobial effect. This differential activity is attributed to eugenol’s phenolic structure, high lipophilicity, and its ability to disrupt bacterial membranes. Notably, pure eugenol and estragole displayed greater antibacterial activity than their crude essential oils, likely due to the absence of antagonistic minor components. Complementary *in silico* evaluations revealed that both compounds possess acceptable drug-likeness and favorable ADME profiles, with eugenol demonstrating enhanced oral bioavailability, membrane permeability, and stronger compliance with Lipinski’s Rule of Five and drug-likeness databases such as CMC and WDI. Additionally, molecular docking simulations confirmed that eugenol formed more stable and diverse interactions with bacterial targets, supporting its lead candidacy. However, toxicity predictions flagged potential mutagenic, carcinogenic, and cardiotoxic (hERG inhibition) risks for both ligands, indicating the necessity for structural refinement and toxicity mitigation. Overall, eugenol emerges as a promising natural antibacterial agent with favorable pharmacokinetic and bioactivity profiles, meriting further preclinical studies for its potential therapeutic application.

## Figures and Tables

**Table 1:** The 3D representative Protein Data Bank (PDB) entries for *E. coli*, *S. aureus*, and *P. aeruginosa*.

*E. coli*		
1FJ4	4K3P	5U10
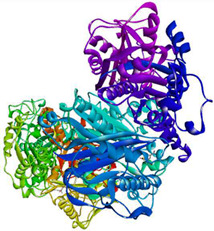	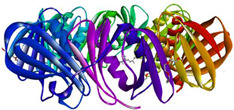	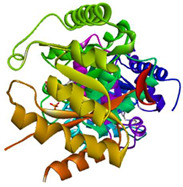
*S. auerus*		
1QME	3T05	3WQT
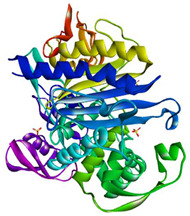	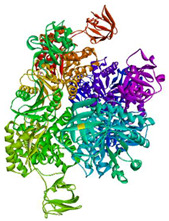	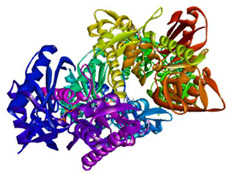
*P. aeruginosa*		
1IUV	1RTT	8AID
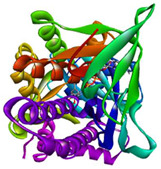	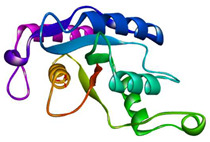	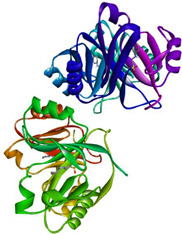

**Table 2:** Results of clove bud essential oil extraction.

Type of Extraction	Yield of EOs	Mass per Unit of Raw Material (g/kg)
Simple hydrodistillation	10% ± 0.29	100 g/kg
Clevenger hydrodistillation	12% ± 0.14	120 g/kg

**Table 3:** Components of clove bud essential oil.

N	Compounds	Area %	RI ^a^	RI ^b^
1	Eugenol	68.51	1361	1356
2	β-Caryophyllene	16.93	1461	1467
3	Caryophyllene oxide	2.09	1530	1523
4	Acetyleugenol	10.35	1590	1581
	Oxygenated monoterpenes	78.86%		
	Sesquiterpene	16.93%		
	Oxygenated sesquiterpenes	2.09%		
	Total	97.88%		

^a^ Retention index on a DB_5_ column, calculated experimentally using alkanes ranging from C_9_ to C_20_. ^b^ Retention index in Adams’ literature for a DB5 column [27].

**Table 4:** Composition of fennel seeds essential oil.

N	Compound	Area %	RI ^a^	RI ^b^
1	Fenchone	3.26	1071	1087
2	Estragole	93.30	1194	1195
3	*Trans*-Anethol	2.86	1281	1285
	Oxygenated monoterpenes	99.42%		
	Total	99.42%		

^a^ Retention index on a DB5 column, experimentally determined using a series of alkanes C_9_–C_20_. ^b^ Retention index in Adams’ literature for a DB5 column [27].

**Table 5:** Antibacterial activity results of essential oil and their main compounds.

	*S. aureus* (mm)	*E. coli* (mm)	*P. aeroginosa* (mm)
Eugenol	16.65 ± 0.66 ^b^	18.65 ± 0.83 ^b^	9.80 ± 0.50 ^b^
Estragole	10.22 ± 0.69 ^d^	10.22 ± 0.38 ^d^	7.77 ± 0.19 ^c^
EO of clove bud	15.00 ± 0.25 ^c^	15.00 ± 0.72 ^c^	11.33 ± 0.44 ^a^
EO of fennel	9.50 ± 0.72 ^e^	9.67 ± 0.45 ^e^	7.00 ± 0.32 ^c^
Amoxicilline 30 μL	22.00 ± 0.33 ^a^	23.00 ± 0.39 ^a^	15.00 ± 0.44 ^a^

Data are expressed as mean ± SD. Statistical significance was determined by Student’s *t*-test and one-way ANOVA (SPSS v20). The letters “a–e”in the table represent groups of results that are statistically compared. These letters indicate that data points with the same letter (for example, two values both marked with “a”) are not significantly different from each other, while those with different letters (such as “a” and “b”) are significantly different.

**Table 6:** Microdilution (Diffusion on liquid medium).

		MIC (mg/mL)				MBC (mg/mL)			
	Samples	Eg	Est	EOC	EOF	Eg	Est	EOC	EOF
Strains									
*S. aureus*		0.58 ^a^	2.30 ^b^	4.07 ^c^	73.61 ^d^	9.20 ^a^	14.50 ^b^	16.31 ^c^	18.57 ^d^
*E. coli*		4.60 ^c^	3.16 ^b^	2.03 ^a^	73.61 ^d^	18.40 ^d^	10.08 ^b^	32.63 ^e^	12.28 ^c^
*P. aeruginosa*		2.32 ^b^	4.60 ^c^	0.50 ^a^	73.61 ^d^	9.20 ^a^	15.23 ^c^	32.63 ^d^	18.40 ^b^

Eg: Eugenol, Est: Estragole, EOC: Essential oil of clove bud, and EOF: Essential oil of fennel seeds. Data are expressed as mean ± SD. Statistical significance was determined by Student’s *t*-test and one-way ANOVA (SPSS v20). The letters “a–e” in the table represent groups of results that are statistically compared. These letters indicate that data points with the same letter (for example, two values both marked with “a”) are not significantly different from each other, while those with different letters (such as “a” and “b”) are significantly different.

**Table 7:** Drug-likeness profiling of eugenol and estragole.

ID	Eugenol	Estragol
CMC_like_Rule	Qualified	Not qualified
CMC_like_Rule_Violation_Fields		Molecular_weight
CMC_like_Rule_Violations	0	1
Lead_like_Rule	Suitable if its binding affinity is greater than 0.1 µM	Suitable if its binding affinity is greater than 0.1 µM
MDDR_like_Rule	Mid-structure	Mid-structure
MDDR_like_Rule_Violation_Fields	No_Rings, No_Rotatable_bonds	No_Rings, No_Rotatable_bonds
MDDR_like_Rule_Violations	2	2
Rule_of_Five	Suitable	Suitable
Rule_of_Five_Violations	0	0
WDI_like_Rule	Out of 90% cutoff	In 90% cutoff
WDI_like_Rule_Violation_Fields	Balaban_index_JX	
WDI_like_Rule_Violations	1	0

“No_Rings” indicates that a molecule lacks cyclic structures, and “No_Rotatable_bonds” signifies the absence of single bonds that allow free rotation between non-ring atoms.

**Table 8:** Predicted ADME and physicochemical properties of eugenol and estragole.

ID	Eugenol	Estragol
BBB	2.25544	1.51179
Buffer_solubility_mg_L	1036.58	680.065
Caco2	46.8865	58.0905
CYP_2C19_inhibition	Inhibitor	Inhibitor
CYP_2C9_inhibition	Inhibitor	Inhibitor
CYP_2D6_inhibition	Non	Non
CYP_2D6_substrate	Weakly	Weakly
CYP_3A4_inhibition	Non	Non
CYP_3A4_substrate	Non	Non
HIA	96.774447	100
MDCK	342.148	172.078
Pgp_inhibition	Non	Non
Plasma_Protein_Binding	100	100
Pure_water_solubility_mg_L	862.745	542.189
Skin_Permeability	−1.31092	−1.09879
SKlogD_value	2.66241	2.80598
SKlogP_value	2.66241	2.80598
SKlogS_buffer	−2.19978	−2.33831
SKlogS_pure	−2.2795	−2.43671

**Table 9:** Toxicity and ecotoxicity predictions for eugenol and estragole.

ID	Eugenol	Estragole
algae_at	0.0567231	0.0677448
Ames_test	mutagen	mutagen
Carcino_Mouse	positive	positive
Carcino_Rat	positive	negative
daphnia_at	0.118703	0.120425
hERG_inhibition	medium_risk	medium_risk
medaka_at	0.0188822	0.0186274
minnow_at	0.0124586	0.0129148
TA100_10RLI	positive	positive
TA100_NA	positive	negative
TA1535_10RLI	positive	negative
TA1535_NA	positive	positive

**Table 10:** Docking scores (kcal/mol) of eugenol and estragole against bacterial protein targets.

Strains	*E. coli*			*S. aureus*			*P. aeruginosa*		
Protein	1FJ4	5U10	4K3P	1QME	3T05	3WQT	8AID	1IUV	1RTT
Eugenol	−6.9	−7.1	−6.9	−7.8	−7.6	−7.7	−7.8	−7.6	−6.7
Estragole	−6.8	−6.5	−6.4	−7.3	−6.6	−7.6	−7.4	−7.2	−6.4

**Table 11:** The 2D Ligand–protein interaction of estragole and eugenol with target proteins.

*E. coli*		
1FJ4	4K3P	5U10
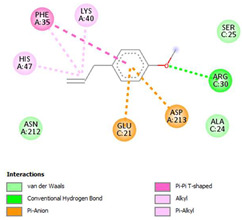	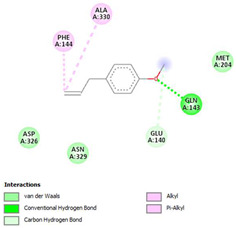	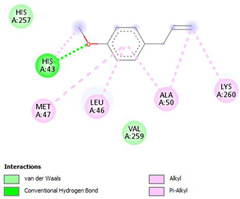
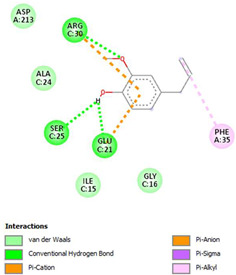	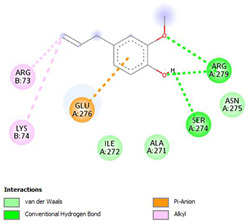	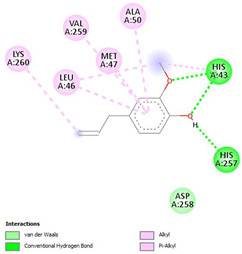
*S. auerus*		
1QME	3T05	3WQT
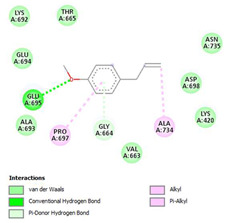	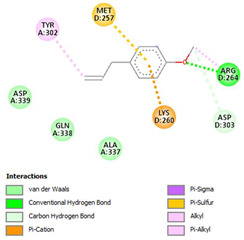	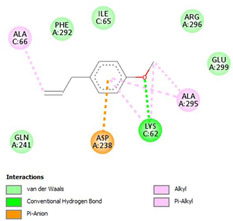
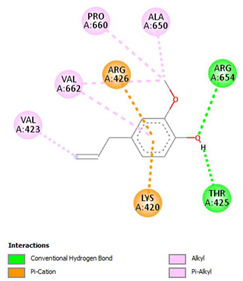	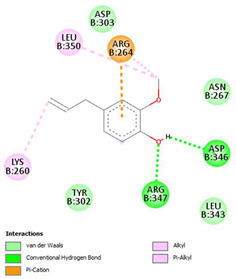	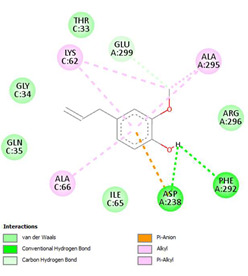
*P. aeruginosa*		
1IUV	1RTT	8AID
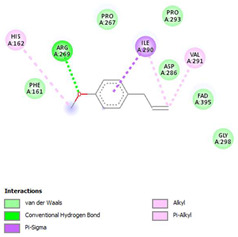	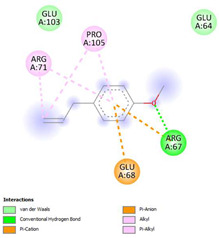	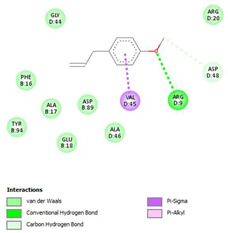
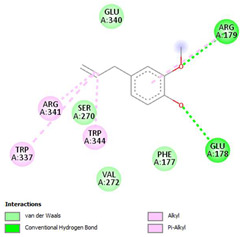	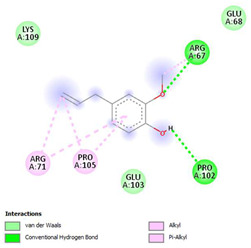	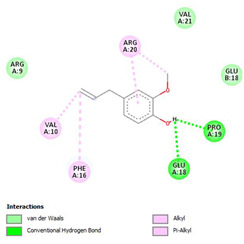

## Data Availability

Data is contained within the article or Supplementary Material: The original contributions presented in this study are included in the article/Supplementary Material. Further inquiries can be directed to the corresponding author(s).

## Supplementary Materials

The following supporting information can be downloaded at: https://www.mdpi.com/article/10.3390/cimb47090694/s1.
