# Supplementary material for: Chemical Composition of Clove and Fennel Seed Essential Oils and a Comparison of Their In Silico and In Vitro Antibacterial Activity with That of Their Main Compounds
Source: Curr Issues Mol Biol. 2025 Aug 27;47(9):694. doi: 10.3390/cimb47090694 (PMC12468443; doi:10.3390/cimb47090694)
Supplement: Supplementary file 1 [file cimb-47-00694-s001.zip › cimb-3826562-supplementary.pdf]

current issues in molecular biology

MDPI

Article

Chemical Composition of Clove and Fennel Seed Essential Oils and a Comparison of Their *In Silico* and *In Vitro* Antibacterial Activity with That of Their Main Compounds

Achraf Abdou [1,*], Fatima Ezzahra Maaghloud [1], Fatima Zahra Kamal [2], Said Rammali [3,4], Alin Ciobica [5,6,7,8], Vasile Burlui [9], Cristina Albert [9,*], Abdelhakim Elmakssoudi [1], Bogdan Novac [10] and Mohamed Dakir [1]

[1] Laboratory of Organic Chemistry, Materials, Electrochemistry, and Environment, Faculty of Sciences Ain Chock, Hassan II University, Casablanca, Morocco; fatimaghloud@gmail.com (F.E.M.); h.elmakssoudi@gmail.com (A.E.); dakir_m@yahoo.fr (M.D.)
[2] Higher Institute of Nursing Professions and Health Technical (ISPITS), Casablanca, Morocco; fatimzahra.kamal@gmail.com
[3] Laboratory of Agro-Alimentary and Health, Faculty of Sciences and Techniques, Hassan First University of Settat, B.P. 539, Settat 26000, Morocco; said.rammali90@gmail.com
[4] Human Nutrition, Bioacives and Oncogenetics Team, Faculty of Sciences, Moulay Ismail University, Meknes 11201, Morocco
[5] Department of Biology, Faculty of Biology, "Alexandru Ioan Cuza" University of Iași, Carol I Avenue, No. 20A, 700505 Iași, Romania; alin.ciobica@uaic.ro
[6] "Olga Necrasov" Center, Department of Biomedical Research, Romanian Academy, 010071 Iasi, Romania
[7] "Ioan Haulica" Institute, Apollonia University, Pacurari Street 11, 700511 Iași, Romania
[8] CENEMED Platform for Interdisciplinary Research, "Grigore T. Popa" University of Medicine and Pharmacy of Iasi, 16th Universitatii Street, 700115 Iasi, Romania
[9] Clinical Department, Apollonia University, Păcurari Street 11, 700511 Iasi, Romania; secretariat@univapollonia.ro
[10] Faculty of Medicine, University of Medicine and Pharmacy "Grigore T. Popa", 700115 Iasi, Romania; bogdannvc@gmail.com
* Correspondence: achraf.abdou@etu.univh2c.ma (A.A.); albident72@yahoo.com (C.A.)

Academic Editor(s): Name

Received: date
Revised: date
Accepted: date
Published: date

Citation: Achraf, A.; Maaghloud, F.E.; Kamal, F.Z.; Rammali, S.; Ciobica, A.; Burlui, V.; Albert, C.; Elmakssoudi, A.; Novac, B.; Dakir, M. Chemical Composition of Clove and Fennel Seed Essential Oils and a Comparison of Their *In Silico* and *In Vitro* Antibacterial Activity with That of Their Main Compounds. *Curr. Issues Mol. Biol.* **2025**, *47*, x. https://doi.org/10.3390/xxxxx

Copyright: © 2025 by the authors. Submitted for possible open access publication under the terms and conditions of the Creative Commons Attribution (CC BY) license (https://creativecommons.org/licenses/by/4.0/).

Abstract

This study aimed to assess the chemical composition and antibacterial potential of essential oils (EOs) from two plants: clove buds (*Syzygium aromaticum*) and fennel seeds (*Foeniculum vulgare*) EOs. The major compounds, eugenol and estragole, were isolated from these oils and tested against *Escherichia coli*, *Staphylococcus aureus*, and *Pseudomonas aeruginosa*. The EOs were obtained via hydrodistillation and analyzed using Gas Chromatography–Mass Spectrometry (GC-MS). Clove oil was found to be rich in eugenol (68.51%), while fennel seed oil was dominated by estragole (93.30%). Antibacterial activity, assessed by the agar disc diffusion method and supported by MIC/MBC testing, revealed that eugenol exhibited the highest efficacy, with MIC values ranging from 0.58 to 1.15 mg/mL and MBC values from 1.15 to 2.30 mg/mL, particularly against *S. aureus* and *P. aeruginosa*. *In silico* analysis was conducted to evaluate pharmacokinetics, toxicity, and molecular docking interactions. ADME predictions indicated good oral bioavailability and high membrane permeability for both compounds, with eugenol displaying superior solubility and better compliance with Lipinski's Rule of Five. Molecular docking simulations confirmed the antibacterial potential, with eugenol showing stronger binding affinities to bacterial targets (−7.8 kcal/mol), forming more stable and diverse interactions compared to estragole. However, toxicity predictions indicated potential mutagenic, carcinogenic, and cardiotoxic (hERG inhibition) risks for both compounds.

Keywords: clove bud; seed of fennel; eugenol; estragole; antibacterial activity.

- **Eugenol (1)**

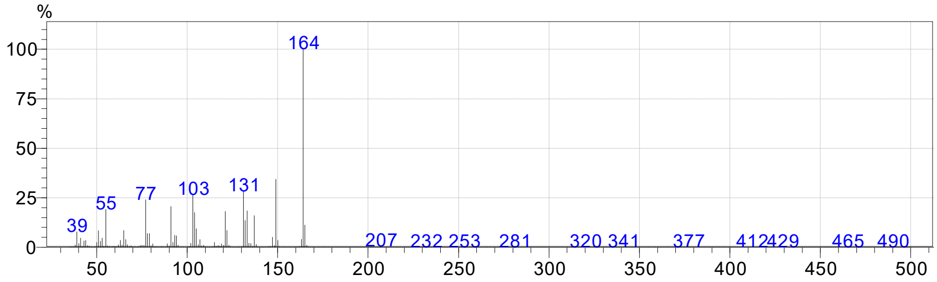

Figure S1. Mass spectrum of eugenol

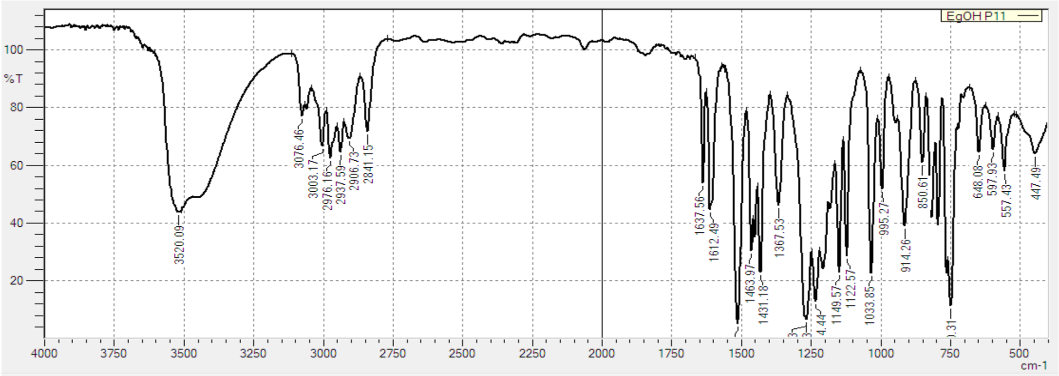

Figure S2. Infra-red spectrum of eugenol

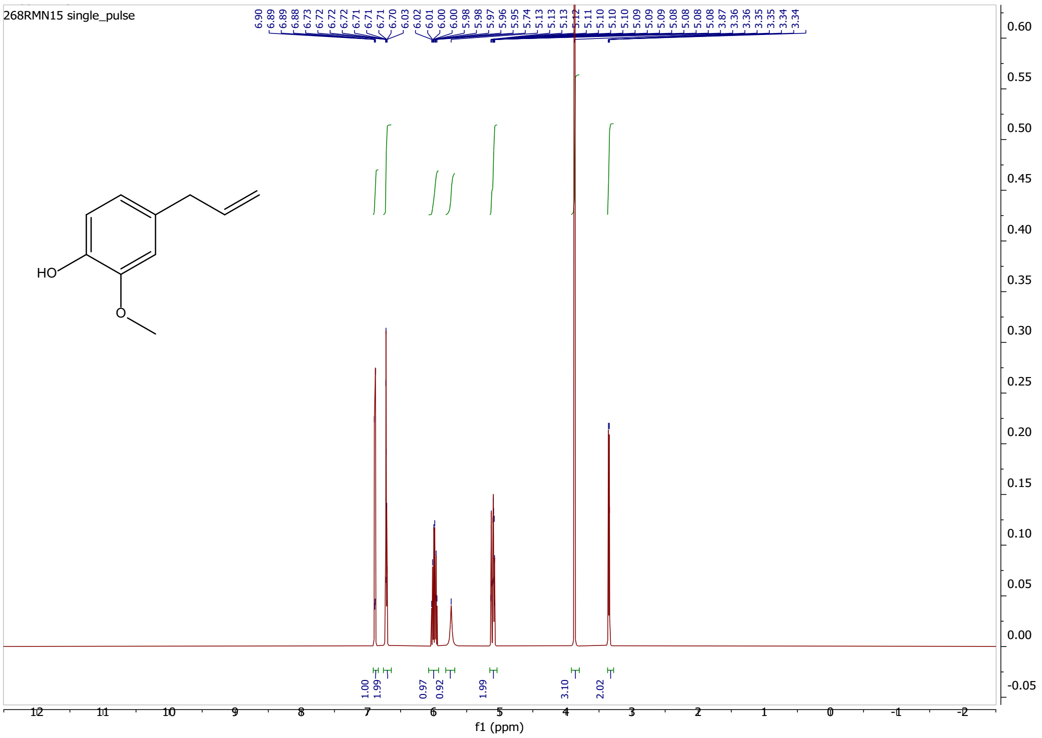

Figure S3. ^{1}H NMR spectrum of eugenol

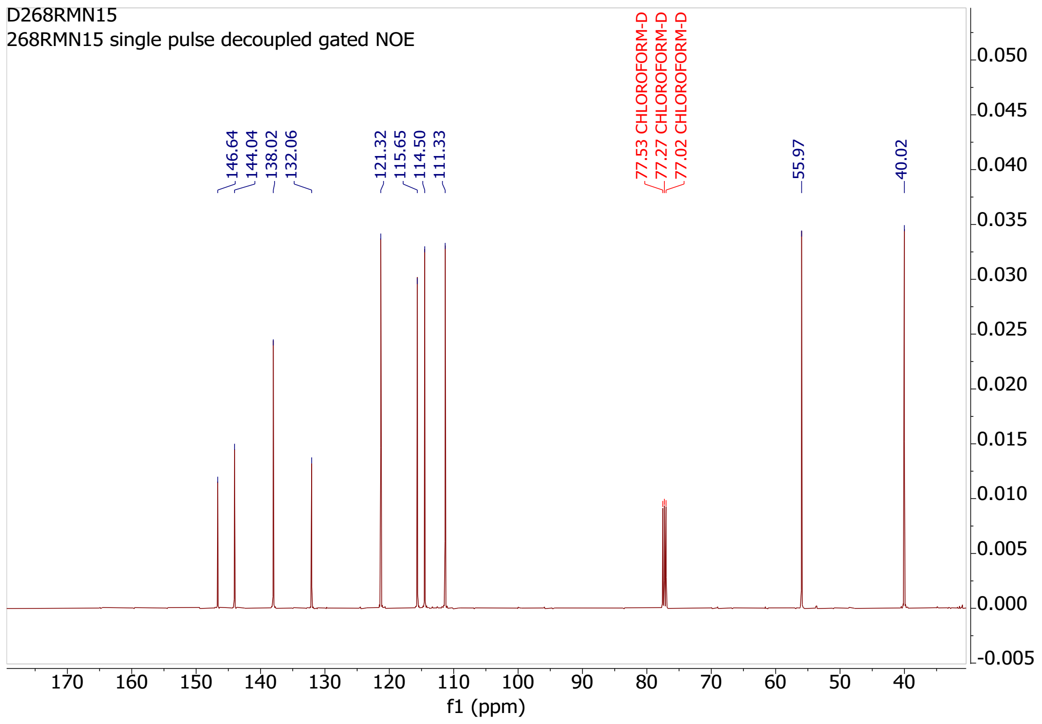

Figure S4. ^{13}C NMR spectrum of eugenol

- **Estragole (2)**

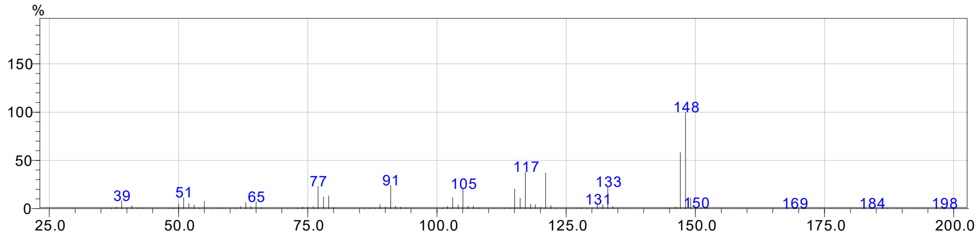

Figure S5. Mass spectrum of estragole

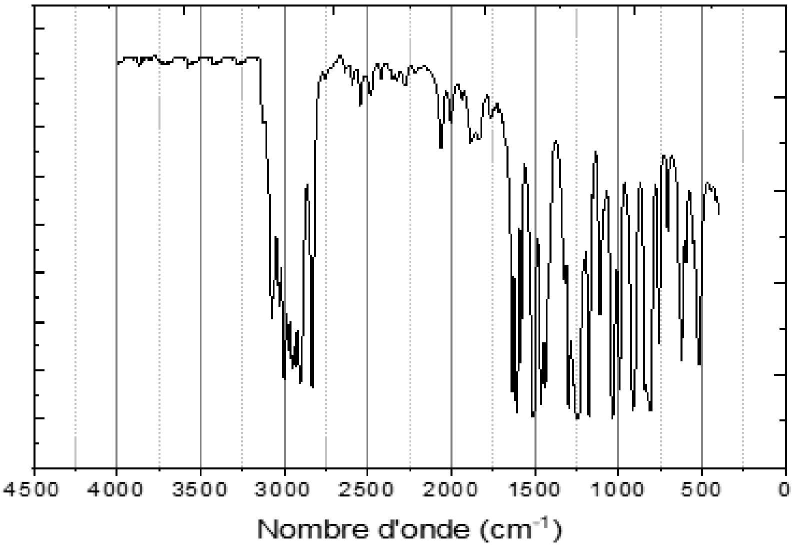

Figure S6. Infra-red spectrum of estragole

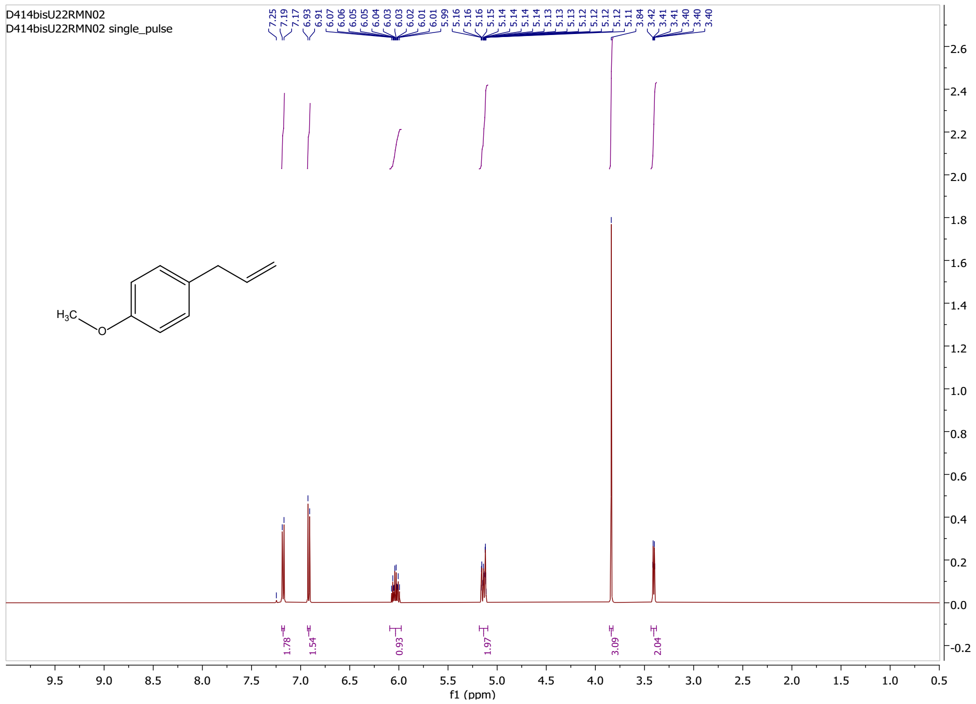

Figure S7. ^{1}H NMR spectrum of estragole

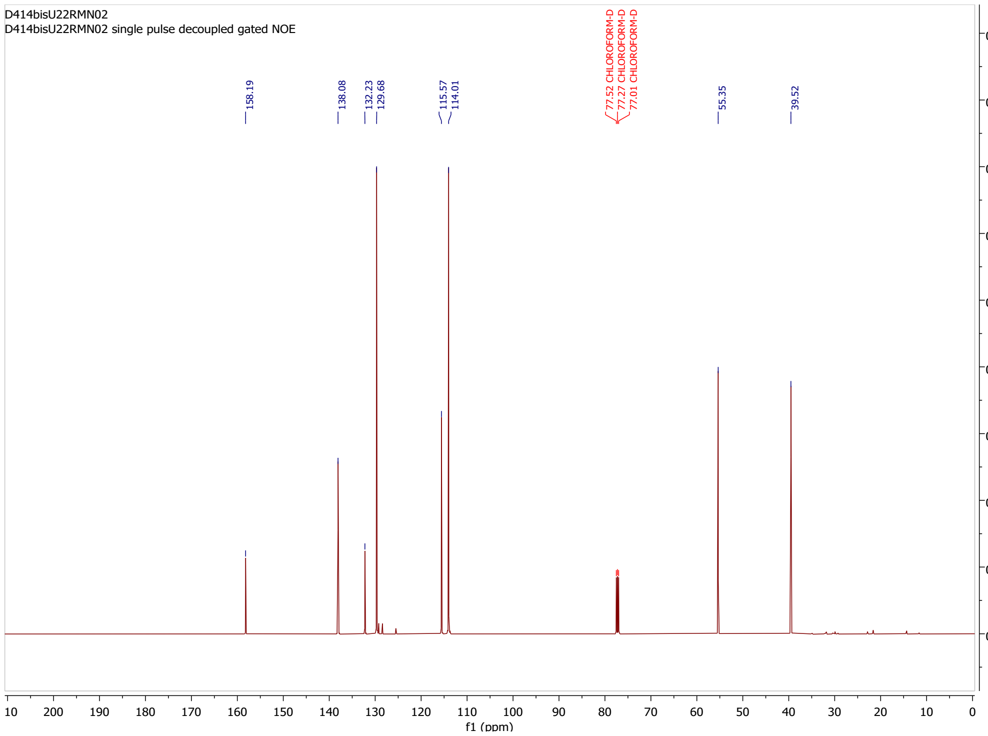

Figure S8. ^{13}C NMR spectrum of estragole
